# Genome of the Parasitoid Wasp *Diachasma alloeum*, an Emerging Model for Ecological Speciation and Transitions to Asexual Reproduction

**DOI:** 10.1093/gbe/evz205

**Published:** 2019-09-25

**Authors:** Eric S Tvedte, Kimberly K O Walden, Kyle E McElroy, John H Werren, Andrew A Forbes, Glen R Hood, John M Logsdon, Jeffrey L Feder, Hugh M Robertson

**Affiliations:** 1 Department of Biology, University of Iowa, IA; 2 Department of Entomology, University of Illinois at Urbana-Champaign, IL; 3 Department of Biology, University of Rochester, NY; 4 Department of Biological Sciences, Wayne State University, Detroit, MI; 5 Department of Biological Sciences, University of Notre Dame, IN; 6 Institute for Genome Sciences, University of Maryland School of Medicine, Baltimore, MD

**Keywords:** Hymenoptera, sequential speciation, de novo genome assembly, genome evolution, chemosensory genes

## Abstract

Parasitoid wasps are among the most speciose animals, yet have relatively few available genomic resources. We report a draft genome assembly of the wasp *Diachasma alloeum* (Hymenoptera: Braconidae), a host-specific parasitoid of the apple maggot fly *Rhagoletis pomonella* (Diptera: Tephritidae), and a developing model for understanding how ecological speciation can “cascade” across trophic levels. Identification of gene content confirmed the overall quality of the draft genome, and we manually annotated ∼400 genes as part of this study, including those involved in oxidative phosphorylation, chemosensation, and reproduction. Through comparisons to model hymenopterans such as the European honeybee *Apis mellifera* and parasitoid wasp *Nasonia vitripennis*, as well as a more closely related braconid parasitoid *Microplitis demolitor*, we identified a proliferation of transposable elements in the genome, an expansion of chemosensory genes in parasitoid wasps, and the maintenance of several key genes with known roles in sexual reproduction and sex determination. The *D. alloeum* genome will provide a valuable resource for comparative genomics studies in Hymenoptera as well as specific investigations into the genomic changes associated with ecological speciation and transitions to asexuality.

## Introduction

The Hymenoptera may be the largest order of insects due to the immense diversity of parasitic wasps (i.e., “parasitoids”) that lay their eggs into or on other insect species (LaSalle and Gauld 1993; Austin and Dowton 2000; Whitfield 2003; Forbes et al. 2018). The great diversity of parasitoid wasps may be a consequence of their close relationship with their insect hosts. When a specialist parasitoid shifts to a new host, this change can propel the evolution of reproductive isolating barriers between wasp populations using the new and ancestral hosts (Feder and Forbes 2010). The evolution of reproductive isolating barriers following a host shift is a well-documented phenomenon in host specialist insects (Forbes et al. 2017), but the study of genomic changes that accompany such phenomena is still in its early stages.

*Diachasma alloeum* (Hymenoptera: Braconidae) is a specialist parasitoid of the fruit fly *Rhagoletis pomonella* (Diptera: Tephritidae). After the introduction of domesticated apples to the United States from Europe, *R. pomonella* infesting native hawthorn fruits experienced a host shift and subsequently evolved reproductive isolating barriers in what has become a well-known example of incipient ecological speciation (Walsh 1867; Bush 1966, 1994; Nosil 2012). This new “apple maggot fly” was sequentially colonized by *D. alloeum*, which appears to have shifted from its ancestral host, the blueberry maggot *Rhagoletis mendax* (Forbes et al. 2009). Two reproductive isolating barriers (i.e., diapause emergence and host fruit volatile discrimination) have evolved in parallel in *R. pomonella* and *D. alloeum*, and in both fly and wasp, these traits appear to have a genetic basis (Dambroski et al. 2005; Forbes and Feder 2006; Forbes et al. 2009). This phenomenon of “sequential” or “cascading” speciation may be an important driver of new biodiversity (Stireman et al. 2006; Abrahamson and Blair 2007; Hood et al. 2015).

Reproductive isolation in genus *Diachasma* has also arisen as a consequence of the loss of sexual reproduction, a general pattern observed in many hymenopteran insects (van der Kooi et al. 2017; Tvedte et al. 2019). Asexual *Diachasmamuliebre* appears to have split from its sexual sister *Diachasmaferrugineum* between 0.5 and 1 Ma (Wharton and Marsh 1978; Forbes et al. 2013). Although the decay of genes involved in sexual traits has been observed in multiple asexual parasitoid wasps (Ma et al. 2014; Kraaijeveld et al. 2016), there is a lack of comparative assessments of genomic molecular evolution between sexual and asexual Hymenoptera.

Here, we report the de novo genome assembly of the parasitoid wasp *D. alloeum*, adding to the genomic resources for parasitoid wasps, which are underrepresented among available hymenopteran genomes (Branstetter et al. 2018). We performed a series of descriptive analyses to assess the overall quality and content of the *D. alloeum* genome, and then focused on annotation and evolutionary analyses of gene families with potential relevance to speciation and sex determination in *Diachasma*.

## Materials and Methods

We isolated genomic DNA from wasps collected in Fennville, MI. Illumina paired-end, mate pair, and TruSeq Synthetic Long Read (TSLR) libraries were sequenced on an Illumina HiSeq2000. The library from a single haploid male enabled the initial contig assembly, and pooled samples were required to achieve the minimum DNA mass needed for other library preparations. Paired-end and mate pair reads were de novo assembled using SOAPdenovo2 v2.04 (Luo et al. 2012) and TSLR “reads” were added using PBJelly v2 (English et al. 2012). We removed putative microbial contaminant sequences from the assembly that were identified by both BlobTools (Laetsch and Blaxter 2017) and a separate custom pipeline developed by Wheeler et al. (2013) and modified as described in Poynton et al. (2018). We separately assembled the mitochondrial genome de novo using NOVOplasty v2.6.3 (Dierckxsens et al. 2017).

We used ten wasps of each sex to generate two (pooled male and pooled female) paired-end RNASeq libraries and sequenced read libraries using an Illumina HiSeq2500. The input DNA required for library preparation precluded the use of the same biological samples for genome and transcriptome sequencing runs. We combined read data sets and assembled a transcriptome de novo with Trinity (Release April 13, 2014) (http://trinityrnaseq.github.io/; last accessed May 2015) (Grabherr et al. 2011; Haas et al. 2013). Annotation of the *D. alloeum* genome assembly was performed by the NCBI using their Eukaryotic Genome Annotation Pipeline (https://www.ncbi.nlm.nih.gov/genome/annotation_euk/process/; last accessed July 2019), with experimental support from the RNAseq and transcriptome. Manual annotations were added to a *D. alloeum* project on the i5k workspace (https://apollo.nal.usda.gov/diaall/jbrowse/; last accessed May 2018; Poelchau et al. 2015). See Supplementary Material online for additional information on genome sequencing, assembly, and annotation.

## Results and Discussion

### Quality Assessment of Genome Assembly

Libraries from a combination of single and pooled wasp samples contained 182.88 Gb total sequence data. The de novo genome assembly Dall1.0 (GenBank accession: GCA_001412515.1) had 3,968 scaffolds with a total scaffold length of 388.8 Mb and a scaffold N50 of 645,583 bp (supplementary table S1, Supplementary Material online). The presence of prokaryotic-like sequences in eukaryotic genome projects may reflect contamination in sequencing libraries or an actual association between microorganisms and hosts. Of the *D. alloeum* scaffolds, we annotated 656 as likely bacterial contaminants and an additional scaffold (Dall2.0 RefSeq accession: NW_021680771.1) as an apparent lateral gene transfer event from a *Rickettsia* species (see Supplementary Material online). The likely bacterial contaminating scaffolds were removed from the *D. alloeum* assembly, and the assembly containing the remaining 3,313 scaffolds is available as Dall2.0 (GCA_001412515.3).

**Fig. 1.**
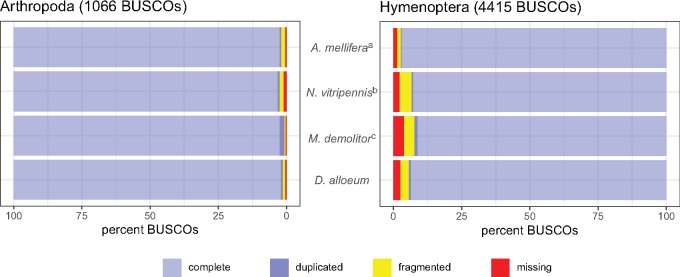
—BUSCO analysis of *Diachasma alloeum* and additional hymenopteran genome assemblies. ^a^*Apis mellifera* assembly reported in Weinstock et al. (2006). ^b^*Nasonia vitripennis* assembly reported in Werren et al. (2010). ^c^*Microplitis demolitor* assembly reported in Burke et al. (2018).

A common metric used to assess the relative completeness of a genome assembly is the identification of conserved single-copy genes, performed here using BUSCO v3 (Simão et al. 2015). We found 1,059/1,066 (99%) Arthropoda BUSCOs and 4,300/4,415 (97%) Hymenoptera BUSCOs in the *D. alloeum* genome, most of which were complete and single-copy (fig. 1). These values are similar to BUSCO gene content in other published hymenopteran genomes, including *Apis mellifera*, *Nasonia vitripennis*, and *Microplitis demolitor* (fig. 1 and see Supplementary Material online). Our de novo assembly of the *D. alloeum* mitochondrial sequence using NOVOplasty (Dierckxsens et al. 2017) produced a 15,936 bp sequence with a complete set of 13 protein coding genes, two rRNA sequences, and 20 tRNA sequences (GenBank accession NW_021683654.1). In addition, our annotation of 65/68 (96%) of the canonical suite of nuclear-encoded mitochondrial genes provided additional evidence for a high-quality genome assembly (see Supplementary Material online).

We used RepeatModeler (Smit et al. 2015), PASTEClassifier (Hoede et al. 2014, version 1.0), and RepeatMasker (Smit et al. 2010) for de novo repeat identification, repeat reclassification, and repeat quantification, respectively (see Supplementary Material online). Remarkably, nearly half (49%) of the *D. alloeum* genome consisted of repetitive sequences, although a substantial contributor (30%) was from unclassified repetitive sequences.

### Chemosensory Gene Repertoire in *D. alloeum*

Chemoreception in arthropods is mediated by three major families of receptors: odorant receptors (ORs), gustatory receptors (GRs), and ionotropic receptors (IRs) (Clyne et al. 1999, 2000; Benton et al. 2009). In addition, two major families of water-soluble proteins are responsible for transport and/or quenching of ligands to chemosensory receptors: odorant binding proteins (OBPs) and chemosensory proteins (CSPs) (Vieira and Rozas 2011; Pelosi et al. 2014; Larter et al. 2016). Chemosensory discrimination of fruit volatiles is an important axis of divergence among host fly-associated populations of *D. alloeum*, initiating reproductive isolating barriers between these wasps (Forbes et al. 2009).

Previous characterizations of chemosensory genes in hymenopteran insects, in particular the gene-rich receptor families, demonstrate that automated gene prediction pipelines are generally poor at accurately predicting these gene models (Robertson and Wanner 2006; Croset et al. 2010; Robertson et al. 2010, 2018; Zhou et al. 2015). We therefore manually annotated a total of 321 gene models that represents the full inventory of five chemosensory gene families in *D. alloeum* (table 1 and see Supplementary Material online). The OR, GR, and IR gene families were larger in *D. alloeum* and other parasitoid wasps relative to *A. mellifera.* We found *D. alloeum* OR lineages in addition to clusters of GRs present in the braconid wasps *D. alloeum* and *M. demolitor* but absent in the well-studied hymenopterans *N. vitripennis* or *A. mellifera* (see Supplementary Material online). We also observed an increased number of IRs in *D. alloeum* relative to another *Microplitis* species, *M. mediator* (see Supplementary Material online). Although we identified chemosensory gene clusters specific to *D. alloeum*, the extensive gene duplication, gene loss, and sequence divergence in these families resulted in poor phylogenetic resolution and indeterminate orthology between gene family members. The difficulty in attributing gene expansions to *D. alloeum* is compounded by the relative lack of genome resources for parasitoid wasps.

**Table 1:** Chemosensory Gene Content of Selected Hymenopteran Insects

Organism	ORs	GRs	IRs	OBPs	CSPs	Citations
*Diachasma alloeum*	187 (14)	39(1)	51 (5)	15 (0)	9 (0)	This study
*Apis mellifera*	163 (11)	10(0)	10 (0^a^)	21 (0^a^)	6 (0^a^)	Robertson and Wanner (2006); Forêt and Maleszka (2006); Forêt et al. (2007); Croset et al. (2010); Elsik et al. (2014)
*Nasonia vitripennis*	225 (76)	47(11)	99(54)	90 (8)	9 (0)	Robertson et al. (2010); Robertson et al. (2018); Werren et al. (2010); Vieira et al. (2012)
*Microplitis demolitor* ^b^	218 (4)	85(1)				Zhou et al. (2015)
*Microplitis mediator*			17 (0^a^)	20 (0^a^)	3 (0^a^)	Zhang et al. (2009); Wang et al. (2016); Peng et al. (2017)

Note.—Intact gene counts are outside parentheses and pseudogene counts are inside parentheses.

a Pseudogene counts were not addressed explicitly in the study.

b Zhou et al. (2015) provided counts of truncated models and pseudogenes for ORs and GRs, however, these sequences were not published and therefore were not used in building phylogenies.

In summary, this gene set is an important resource for future studies of the evolutionary history of *Diachasma* chemosensory genes. It will be critical to ascertain the members of the *D. alloeum* chemosensory repertoire that operate specifically in chemosensory behavior. Although the families are generally well conserved across insects, the challenge of orthology assessment and the limited functional study of these genes make it difficult to estimate the precise chemosensory inventory of *D. alloeum*. ORs operate specifically in odorant recognition, and the expansion of OR genes in insects may have been adaptive during the transition to terrestrial life (Robertson et al. 2003, but see Missbach et al. 2014). Although relatively understudied, the IR family has a likely protostome origin, and conservation of multiple orthologs initially identified in *Drosophilamelanogaster* suggest an important function of IR genes in olfaction across insects (Rytz et al. 2013). Conversely, the origin of GRs dates back to the Placozoa, and GR-like genes in basal animals function in development, not chemosensation (Robertson 2015; Saina et al. 2015). The OBP and CSP transporter families have roles in chemical ligand delivery to chemosensory receptors but also function in release of pheromones, reproductive processes, and embryonic development (Pelosi et al. 2018). Transcriptome data sets used for *D. alloeum* gene predictions were taken from pooled whole male and female wasps, so we cannot exclude the possibility that some genes have nonchemosensory roles. Future studies should incorporate tissue-specific RNA data sets to provide stronger support for genetic components of chemosensation in *D. alloeum*.

Chemosensory genes are promising candidates for differential selective regimes in apple and hawthorn populations of *D. alloeum*. *Rhagoletis pomonella* host flies use olfactory cues from ripening fruit to identify suitable sites for mating and oviposition (Linn et al. 2003). Like *R. pomonella*, *D. alloeum* parasitoids have demonstrated odor preferences for their host fruits, representing a potential prezygotic reproductive barrier preventing mating between wasp populations utilizing different hosts (Forbes et al. 2009). Evolutionary rate and differential expression analyses of chemosensory genes in *D. alloeum* populations could be potential areas of inquiry.

Chemosensory gene evolution could also be influenced by transitions in reproductive strategies in *Diachasma*. Wasp courtship is mediated by the male perception of sex pheromones produced by females (Boush and Baerwald 1967). Across arthropods, chemosensory genes demonstrate differential expression in males and females (Zhou et al. 2012; Shiao et al. 2013; Eyun et al. 2017). Chemosensory genes showing strong sex bias may be candidates for degradation in an asexual genome, such as those involved in female signaling or male recognition of mate signals (Normark et al. 2003; Tabata et al. 2017). Future studies could assess sex-specific expression of chemosensory genes in *D. alloeum* and corresponding evolutionary patterns in its asexual relative *D. muliebre*.

### *Diachasma a* *lloeum* Contains Canonical Genes Involved in Reproduction and Sex Determination

Hymenoptera is an insect order characterized by haplodiploid sex determination, providing an opportunity for studying the evolution of reproductive modes, including transitions from sexual to asexual systems. Meiosis is essential to obligate sexual reproduction, such that loss of sex may be accompanied by the subsequent degradation of meiotic genetic machinery (Schurko and Logsdon 2008). However, identical sets of meiosis genes in *D. alloeum* (sexual) and *D. muliebre* (asexual) (Tvedte et al. 2017) and population genetic data implying that the asexual *D. muliebre* undergoes recombination (Forbes et al. 2013) together suggests that asexual wasps retain meiotic production of gametes despite the loss of sexual reproduction. Given the apparent lack of male production in *D. muliebre*, a noncanonical form of meiosis could facilitate the maintenance of genetic variation and promote the persistence of this asexual lineage.

In many hymenopterans, development into male versus female forms is based on allelic states at a single locus, a mechanism known as complementary sex determination (CSD) (van Wilgenburg et al. 2006). In *A. mellifera* specifically, sex determination depends on the *csd* gene (Hasselmann et al. 2008). We found no evidence of the *csd* locus in *D. alloeum*, however our inability to consistently rear wasps in the laboratory at the current time precludes our ability to definitively rule out CSD as a sex determination mechanism. In CSD and non-CSD hymenopterans, a well-conserved sex determination regulatory cascade includes *transformer* and *doublesex*, both displaying sex-specific splicing (Geuverink and Beukeboom 2014). We annotated male and female isoforms of *transformer* and *doublesex* genes in *D. alloeum* (GenBank accessions THK33055.1, THK33056.1, THK32977.1, THK32978.1).

Sex determination genes may be targets of selection in asexual Hymenoptera. Across insects, male production occurs due to alternative splicing of *transformer* rendering the protein nonfunctional, leading to male-splicing of *doublesex*. Conversely, translation of full-length *transformer* into functional protein mediates the splicing of female-specific *doublesex* isoforms (Verhulst et al. 2010). RNA-seq read mapping patterns supported sex-specific *transformer* isoforms in *D. alloeum* (see Supplementary Material online). In all-female *Diachasma* species, we would expect selection to preserve the full-length *transformer* gene. In *doublesex*, the female isoform in *D. alloeum* is shorter (see Supplementary Material online), similar to splicing patterns in other insects (Cho et al. 2007; Oliveira et al. 2009). The single exon specific to males may be subject to future degradation following sex loss in asexual *Diachasma* species.

Additional genes contributing to sex-specific traits (e.g., sperm production, pheromones, pigmentation) may be candidates for degradation in asexual wasps (van der Kooi and Schwander 2014; Kraaijeveld et al. 2016). The high quality of *D. alloeum* assembly provides a suitable framework for future studies of the effects of sexual and asexual reproductive modes on patterns of molecular evolution across the wasp genome. 

## Supplementary Material

Supplementary data are available at *Genome Biology and Evolution* online.

## Supplementary Material

evz205_Supplementary_Data

## Notes

**Data deposition:** This project has been deposited at GenBank under the accessions PRJNA284396 (whole genome) and PRJNA283787 (transcriptome).
